# Evolution of Vertebrate Transient Receptor Potential Vanilloid 3 Channels: Opposite Temperature Sensitivity between Mammals and Western Clawed Frogs

**DOI:** 10.1371/journal.pgen.1002041

**Published:** 2011-04-07

**Authors:** Shigeru Saito, Naomi Fukuta, Ryuzo Shingai, Makoto Tominaga

**Affiliations:** 1 Division of Cell Signaling, Okazaki Institute for Integrative Bioscience (National Institute for Physiological Sciences), National Institutes of Natural Sciences, Okazaki, Aichi, Japan; 2 Laboratory of Bioscience, Faculty of Engineering, Iwate University, Morioka, Iwate, Japan; 3 Department of Physiological Sciences, The Graduate University for Advanced Studies, Okazaki, Aichi, Japan; University of Michigan, United States of America

## Abstract

Transient Receptor Potential (TRP) channels serve as temperature receptors in a wide variety of animals and must have played crucial roles in thermal adaptation. The *TRP vanilloid* (*TRPV*) subfamily contains several temperature receptors with different temperature sensitivities. The TRPV3 channel is known to be highly expressed in skin, where it is activated by warm temperatures and serves as a sensor to detect ambient temperatures near the body temperature of homeothermic animals such as mammals. Here we performed comprehensive comparative analyses of the *TRPV* subfamily in order to understand the evolutionary process; we identified novel *TRPV* genes and also characterized the evolutionary flexibility of *TRPV3* during vertebrate evolution. We cloned the TRPV3 channel from the western clawed frog *Xenopus tropicalis* to understand the functional evolution of the TRPV3 channel. The amino acid sequences of the N- and C-terminal regions of the TRPV3 channel were highly diversified from those of other terrestrial vertebrate TRPV3 channels, although central portions were well conserved. In a heterologous expression system, several mammalian TRPV3 agonists did not activate the TRPV3 channel of the western clawed frog. Moreover, the frog TRPV3 channel did not respond to heat stimuli, instead it was activated by cold temperatures. Temperature thresholds for activation were about 16 °C, slightly below the lower temperature limit for the western clawed frog. Given that the TRPV3 channel is expressed in skin, its likely role is to detect noxious cold temperatures. Thus, the western clawed frog and mammals acquired opposite temperature sensitivity of the TRPV3 channel in order to detect environmental temperatures suitable for their respective species, indicating that temperature receptors can dynamically change properties to adapt to different thermal environments during evolution.

## Author Summary

Evolution of temperature perception is crucial for adaptation to thermal environments; however, this process is poorly understood. Here we investigated the evolution of the vertebrate *TRPV* subfamily which contains several mammalian temperature receptors. We identified several novel *TRPV* genes that have not been found previously and discovered evolutionary flexibility of the *TRPV3* gene during vertebrate evolution. TRPV3 channels perceive warm temperature and serve as sensors to detect ambient temperatures near the body temperature of homeothermic animals such as mammals. To examine the functional evolution of TRPV3 channels in vertebrate evolution, we cloned the gene from the western clawed frog and found that its N- and C-terminal regions were highly diversified from those of other terrestrial vertebrate TRPV3 channels. Characterization of the channel properties of western clawed frog TRPV3 revealed that it was not activated by heat stimuli, but instead was activated by cold stimuli. Temperature thresholds for activation were about 16 °C, slightly below the lower temperature limit for the western clawed frog. Thus, the western clawed frog and mammals acquired opposite temperature sensitivity of TRPV3 channels to detect environmental temperatures suitable for their respective species, indicating that temperature receptors can dynamically change properties to adapt to thermal environments during evolution.

## Introduction

Animals adapt to environmental temperature changes by sensing both their body and ambient temperatures. Thermal stimuli are detected by temperature receptors and transmitted by the peripheral nerves in which they reside [1]–[4]. Thus, temperature receptors must have served crucial roles in adaptation to thermal environments during the course of evolution. In mammals, temperature receptors are ion channels that are activated by thermal stimuli [1]–[4]. In humans and rodents, nine temperature receptors have currently been identified and all of them belong to the transient receptor potential (TRP) cation channel superfamily and are called “thermoTRPs”. These nine thermoTRPs are further classified into three subfamilies: four belong to the TRP vanilloid subfamily (*TRPV1-TRPV4*), four to the TRP melastatin subfamily (*TRPM2*, *TMPM4*, *TRPM5*, and *TRPM8*) and one to the TRP ankyrin subfamily (*TRPA1*) [1], [3]. Phylogenetic analysis of vertebrate thermoTRP homologs revealed that the genes encoding TRPV1-TRPV4, TRPM2, TRPM4, TRPM5, and TRPM8 are unique to vertebrates [5]. Most of these genes emerged in the common ancestor of teleost fishes and terrestrial vertebrates through repeated gene duplications; subsequent sequence divergence resulted in a thermoTRP repertoire with different physiological properties. In humans and rodents, TRPV1 and TRPV2 are activated by noxious high temperatures, TRPM8 and TRPA1 by cold temperatures, and TRPV3, TRPV4, TRPM2, TRPM4, and TRPM5 by warm temperatures [1]–[4]. In addition to thermal stimuli, thermoTRPs are also activated by various physical and chemical stimuli [1]–[4]. Thus, thermoTRPs are involved in various sensory transductions and required for the adaptation to ambient environments.

TRPV3 and TRPV4 perceive warm temperatures in homeothermic animals such as mammals [6]–[8] and thus must play important roles in body temperature regulation. Consistent with this idea, *TRPV3* knockout mice showed abnormalities in sensing ambient temperatures near their body temperature [9]. However, whether these warm-temperature receptors are also physiologically important for the ectothermic vertebrates remains unknown. With respect to the *TRPV4* gene, all of the vertebrate species thus far examined possess *TRPV4* orthologs with highly conserved amino acid sequences among these different vertebrate species [5]. Regarding the *TRPV3* gene, although all terrestrial vertebrate species thus far examined possess one copy of a *TRPV3* orthologous gene, it has been lost in two teleost fish species [5]. Additionally, in the genome sequence database of the western clawed frog *Xenopus tropicalis* (belonging to the amphibian class) [10], the predicted gene for *TRPV3* is much shorter than mammalian orthologs due to the lack of the N- and C-terminal portions. Sequences homologous to the mammalian terminal regions were searched, but such regions have not been found in the genome sequence of the western clawed frog [5]. This implies that the N- and C-terminal regions of western clawed frog TRPV3 have diverged from those of the mammalian orthologs, thus the complete coding sequence of the *TRPV3* gene could not be annotated bioinformatically utilizing mammalian *TRPV3s*. Since amino acid sequences of the terminal regions of TRPV3s are well conserved among several mammalian species, divergence of TRPV3 may reflect a functional shift of the TRPV3 channel between mammals and the western clawed frog. Thus TRPV3 is a suitable model for understanding how thermoTRPs have changed their amino acid sequences as well as function during the course of evolution. Moreover, comparison of TRPV3 channel properties between homeothermic and ectothermic vertebrates may supply new insights into the functional evolution of thermoTRPs related to body temperature differences among species.

In contrast to the well characterized TRPV3 channels in homeothermic animals such as mammals, information on the TRPV3 channel in ectothermic animals is quite limited. Grandl *et al*. (2008) cloned western clawed frog *TRPV3* (which lacked the N- and C-terminal regions) into a mammalian expression vector [11], but this truncated TRPV3 was nonfunctional. They subsequently fused the N- and C-terminal regions of mouse TRPV3 to western clawed frog TRPV3 and found that this chimeric TRPV3 responded to heat, camphor, and 2-aminoethoxydiphenyl borate (2-APB) [11], [12], known activators of mammalian TRPV3 in cultured mammalian cells [6]–[9], [13], [14]. However, since these observations were obtained using chimeric TRPV3, they did not show the native channel properties of western clawed frog TRPV3. In order to characterize the amino acid sequence as well as the channel properties of native TRPV3 from the western clawed frog, determination of the entire cDNA sequence is necessary.

The aim of the present study is to understand evolutionary changes in the TRPV3 channels. In this study, we sequenced cDNA of western clawed frog *TRPV3* including the 5′- and 3′-untranslated regions (UTR) and compared the amino acid sequences among various terrestrial vertebrate species. To characterize its channel properties, we cloned *TRPV3* into an expression vector and used a heterologous expression system to compare properties between mammals and amphibians. Additionally, we conducted comprehensive comparative and phylogenetic analyses of the vertebrate *TRPV* subfamily utilizing various genome sequence databases to elucidate the evolutionary processes that occurred within the vertebrate lineages. Here we report the evolutionary changes of the TRPV3 channels, and highlight the differences in the temperature sensitivities between mammals and anurans.

## Results

### Reconstruction of the vertebrate TRPV subfamily phylogenetic tree

**Figure 1:**
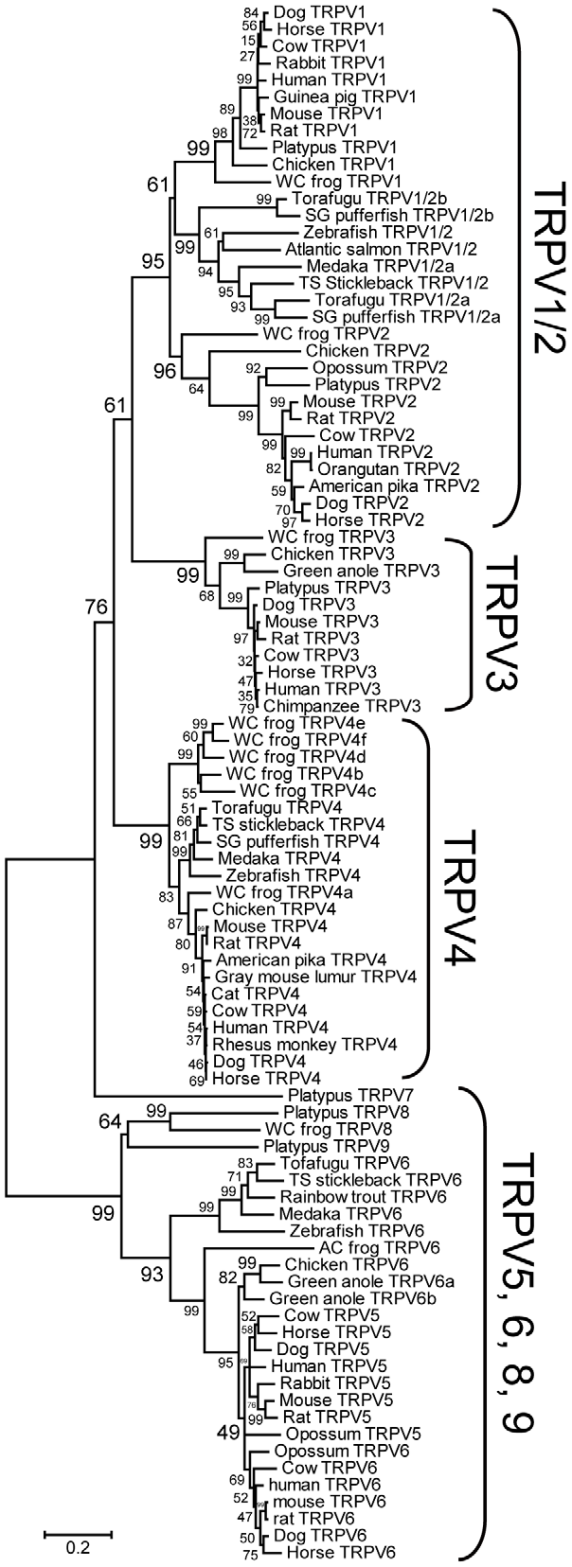
Phylogenetic relationship of the *TRPV* gene subfamily of vertebrates. Statistical confidence (bootstrap value) is indicated beside the respective branch [15]. The *TRPV5/6* genes were used as outgroups. WC frog, AC frog, SG pufferfish, and TS stickleback indicate western clawed frog, African clawed frog, spotted green pufferfish, and three-spined stickleback, respectively.

In order to understand the evolutionary changes within the TRPV subfamily, a comprehensive phylogenetic tree containing a broad range of vertebrate species including mammals, chicken, green anole, western clawed frog, and teleost fishes was reconstructed (Figure 1) [15]–[17]. The *TRPV5* and *TRPV6* genes that code for non-temperature-sensitive channels first diverged from the *TRPV1-TRPV4* genes. Among the *TRPV1-4* genes, each member was monophyletic to each other with high bootstrap value (>96%). The *TRPV4* cluster was first to diverge, followed by a split of the *TRPV3* cluster from the vertebrate *TRPV1/2* cluster. However, the order of divergence between the *TRPV3* and *TRPV4* clusters was not clearly resolved since the connection between the vertebrate *TRPV1/2* and *TRPV3* clusters was supported only by a moderate bootstrap value (61%). Within the *TRPV1/2* cluster, the terrestrial vertebrate *TRPV2* cluster first split from the clusters containing the teleost fish *TRPV1/2* and terrestrial vertebrate *TRPV1* genes, although this branching order was supported by a moderate bootstrap value (61%). In dog, cow, and horse, one copy each of the *TRPV1-TRPV6* genes were found. In the green anole, one copy each of the *TRPV1-4* genes, and two copies of the *TRPV6* gene were found. Unfortunately, due to low coverage in the green anole genome sequence database, only short sequenced fragments existed for the *TRPV1, TRPV2,* and *TRPV4* genes. Thus, only the *TRPV3* gene and two copies of the *TRPV6* genes were included in the phylogenetic tree (Figure 1). One copy of the *TRPV6* gene (*TRPV6a*) clustered with the chicken *TRPV6* gene; the other copy of *TRPV6* (*TRPV6b*) clustered with the former two genes with high bootstrap value (82%). Thus the gene duplication event which created the two copies of the *TRPV6* gene in the green anole occurred within the reptile/bird lineages independent from the gene duplication event that produced the mammalian *TRPV5* and *TRPV6* genes. This latter duplication event likely occurred within the common ancestor of mammals since opossum also possesses *TRPV5* and *TRPV6* genes.

**Figure 2:**
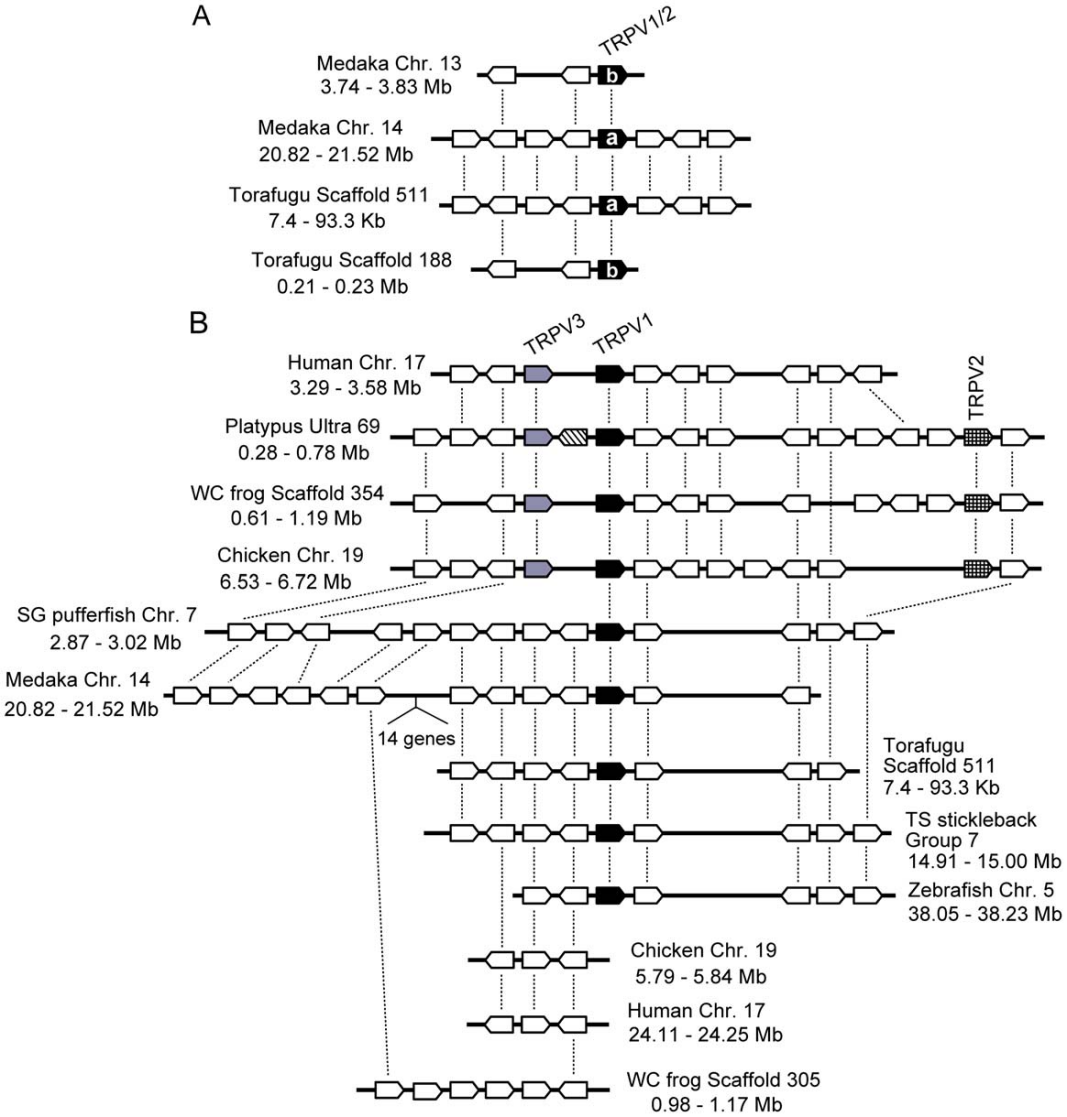
Conserved gene arrangements in the genomic regions encompassing vertebrate *TRPV1-TRPV3* genes. The gene orders around *TRPV1/2* of medaka and torafugu (A) and *TRPV1-TRPV3* of vertebrates (B) are shown. The genes are shown as boxes with their directions indicated. Filled, hatched, gray, and striped boxes represent *TRPV1*, *TRPV2*, *TRPV3*, and *TRPV7* (only found in platypus genome), respectively. The open boxes indicate non-TRP genes. The orthologous genes among different species are connected by lines. The physical distances of the genomic regions are indicated. Chr., chromosome.

Teleost fish *TRPV1/2* genes showed copy number variation among the different species. Zebrafish and three-spined stickleback possessed only one copy, while torafugu, spotted green pufferfish, and medaka possessed two copies (note that the medaka *TRPV1/2b* gene was excluded from the phylogenetic tree in Figure 1 since it has large deletions in the central region which reduces the resolution of the phylogenetic tree; Figure 1 and Figure 2A). The *TRPV1/2a* and *TRPV1/2b* genes in medaka and torafugu were located in different genomic regions in which syntenic relationships were preserved around *TRPV1/2s* (Figure 2A).

The syntenic relationship also existed in the genomic regions around the *TRPV1-TRPV3* genes among vertebrate species (Figure 2B). In platypus, western clawed frog, and chicken, the *TRPV1* and *TRPV3* genes were located adjacently, and *TRPV2* was positioned several genes away from *TRPV1* and *TRPV3*. In humans, the *TRPV1* and *TRPV3* genes were also located adjacently although the *TRPV2* gene was distantly located in the same chromosome [5]. The teleost fish *TRPV1/2* gene was located in a position corresponding to the terrestrial vertebrate *TRPV1* gene (Figure 2B). Although a syntenic relationship can be observed around the *TRPV1-TRPV3* genes among vertebrate species, the genes corresponding to the terrestrial vertebrate *TRPV3* and *TRPV2* were not found in the teleost fish genome sequences (Figure 2B).

**Figure 3:**
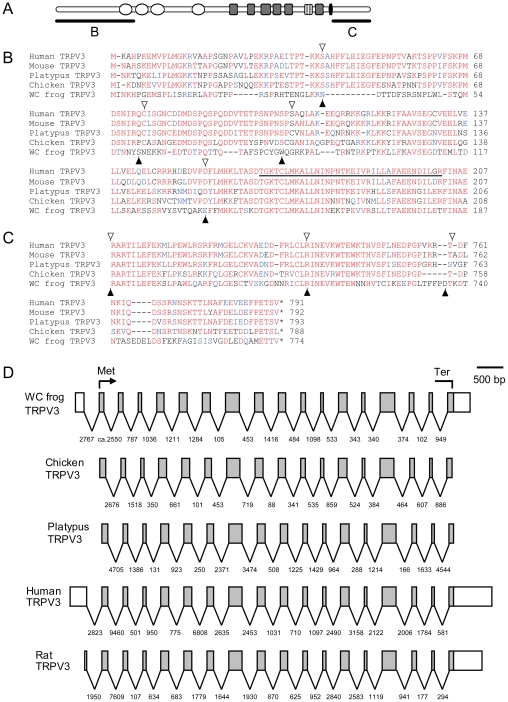
Comparison of TRPV3 among representative terrestrial vertebrate species. The schematic structure of the TRPV3 channel (A) is shown and the N- (B) and C- (C) terminal regions of amino acid alignments are indicated. The open circles, gray boxes, striped boxes, and black box represent the putative ankyrin repeat, transmembrane, pore loop, and TRP domains, respectively. The amino acids identical to, similar to, and different from the consensus residues are indicated in red, blue, and black letters, respectively. Exon boundaries for human, mouse, platypus, and chicken are indicated by open triangles and those for western clawed frog by filled triangles. (B) The first ankyrin repeat domain is underlined. Gene structures of *TRPV3s* in several vertebrate species (D). Exons are indicated by open boxes according to their scale. Introns are indicated by solid lines with their lengths (bp). The open reading frame is indicated by a shaded area with its initiation (Met) and termination (Ter) codons. Gene structures of chicken and platypus were predicted in the genome sequence databases (Ensembl) but not supported by cDNA data thus exons for the 5′- and 3′-UTR regions are not known. Gene structures of human and rat *TRPV3s* are based on full length cDNA nucleotide sequences and that of the western clawed frog *TRPV3* is base on the cDNA nucleotide sequence determined in the present study.

In the course of phylogenetic analysis, we found several novel *TRPV* genes that have not previously been described. We found one novel gene from platypus that formed a sister group to a cluster of vertebrate *TRPV1-TRPV4* genes, but was located outside of them (tentatively named *TRPV7*) (Figure 1). The *TRPV7* gene was flanked by the *TRPV1* and *TRPV3* genes in the platypus genome sequence (Figure 2B). We could not find a corresponding gene in the other vertebrate species examined, including human, mouse, dog, cow, opossum, chicken, western clawed frog, medaka, and zebrafish. The predicted amino acid sequence of platypus TRPV7 possessed the putative ankyrin repeat and six transmembrane domains that are highly conserved among TRP channels. TRPV7 showed 44.2%, 42.7%, and 44.0% amino acid sequence similarity to platypus TRPV1, TRPV2, and TRPV3, respectively, in the central conserved regions (from ankyrin repeat domain 1 to the TRP domain; Figure 3A). In addition to platypus *TRPV7*, we also found three genes that are closely related to the vertebrate *TRPV5* and *TRPV6* genes (Figure 1). Two of them, from platypus and western clawed frog, formed a monophyletic cluster (tentatively named *TRPV8*). Platypus possessed one additional gene that clustered together with the *TRPV8* genes (tentatively named *TRPV9*). The western clawed frog also possessed a *TRPV6* gene that formed a sister group with the African clawed frog *TRPV6*, although the western clawed frog *TRPV6* gene has a large portion that has not been sequenced yet, thus it was excluded from the phylogenetic tree shown in Figure 1.

### Determination of the cDNA sequences of the western clawed frog TRPV3 gene

Detailed comparative analyses from this and previous studies [5] raised the possibility that TRPV3 of the western clawed frog is diversified from that of mammals. However, as mentioned above, the predicted *TRPV3* gene lacks the N- and C-terminal portions as they could not be annotated bioinformatically from the genome sequence database of the western clawed frog. We performed RT-PCR, 3′- and 5′-RACE using total RNA extracted from the toe of the western clawed frog to sequence the cDNA of western clawed frog *TRPV3* from the 5′- to 3′-UTRs to obtain the full length coding sequence. We obtained a 2819-bp cDNA fragment (AB588024) which had a 2319-bp open reading frame (773 amino acid residues) starting near the 5′ end in the 2nd exon and ending in the last exon (Figure S1). Comparison of this cDNA sequence with the genome sequence database of the western clawed frog revealed that the nucleotide sequence corresponding to the second exon (114 bp) did not exist in the database. To confirm the result obtained by 5′-RACE, the cDNA fragment spanning exons 1 to 6 was amplified by RT-PCR. We obtained an approximately 650-bp DNA fragment that contained the second exon (Text S1). We further amplified and sequenced the genomic regions containing the second exon of the *TRPV3* gene (AB588025) and confirmed that the second exon was located within the genomic portions that had yet to be sequenced by the genome sequence project (Text S1 and Figure S2). Therefore, the existence of the second exon was not an artifact.

Comparison of the amino acid sequences of western clawed frog TRPV3 with those of other terrestrial vertebrate orthologs revealed that it possesses conserved motifs such as four ankyrin repeat domains and six transmembrane domains (Figure S3). The amino acid sequences in the central portion of TRPV3 were relatively conserved among the tetrapod species examined. In contrast, amino acid sequences in the N- and C-terminal regions of western clawed frog TRPV3 were highly divergent from those of amniote TRPV3s although the corresponding regions of TRPV3 among amniotes were relatively well conserved (Figure 3B, 3C and Figure S3). In both terminal regions, a large number of amino acid substitutions as well as many gaps existed between western clawed frog and amniotes TRPV3s. The exon-intron structure of the *TRPV3* gene of the western clawed frog was highly similar to those of other vertebrate *TRPV3* genes (Figure 3D). Highly divergent regions in western clawed frog TRPV3 spanned across several exons although exon boundaries were conserved among the vertebrate species compared (Figure 3). This suggests that the divergence of the terminal regions of TRPV3 was not the result of modification of gene structure; rather, the divergence can be attributed to the accumulation of considerable amino acid substitutions.

### Characterization of ion channel properties of TRPV3 in the western clawed frog

**Figure 4:**
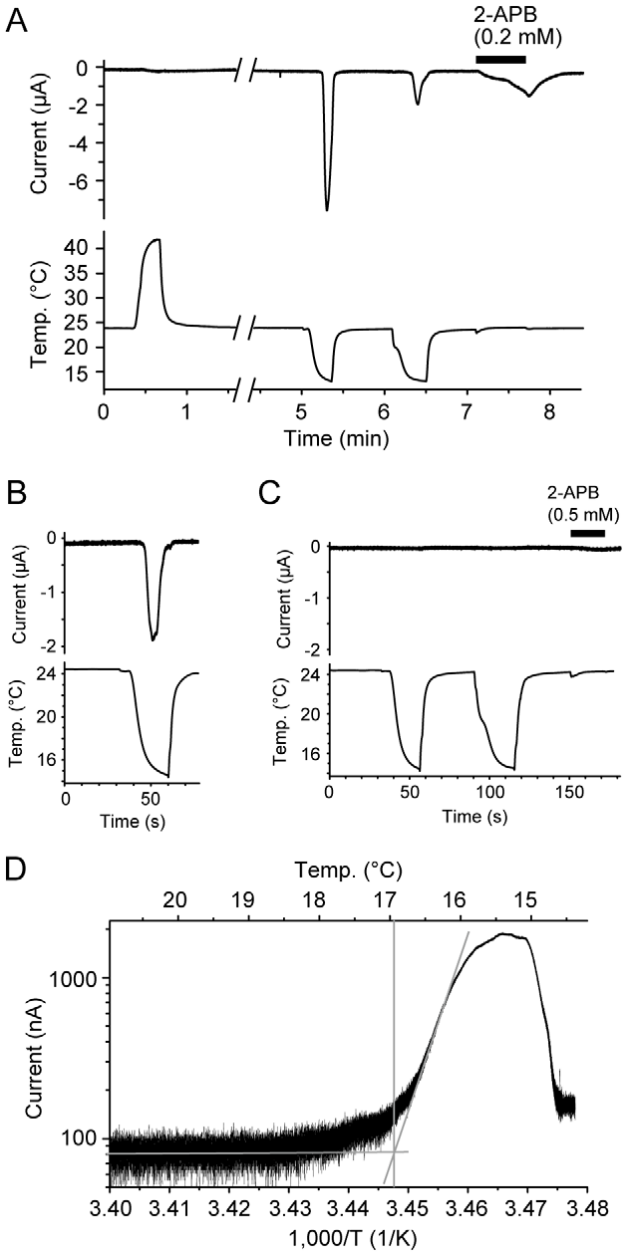
Cold temperature activation of the TRPV3 channel of the western clawed frog. (A and B) Representative current traces (Upper) from oocytes injected with TRPV3 cRNA of the western clawed frog and corresponding temperatures (Lower). The bar indicates the application of 2-ABP (0.2 mM). (C) A representative trace of an oocyte injected with water alone. (D) An Arrhenius plot of the current in panel B. Average temperature threshold for activation was 16.35±0.51°C (n = 14).

We next examined the ion channel properties of TRPV3 in the western clawed frog by expressing it in oocytes of the African clawed frog (*Xenopus laevis*) from which we recorded ionic currents using a two-electrode voltage-clamp method. As the mammalian TRPV3 channel is activated by temperatures >31-39°C [6]-[8], we first asked whether the TRPV3 channel of the western clawed frog is also activated by heat. Heat stimuli, however, did not induce any response in the oocytes injected with western clawed frog complementary RNA (cRNA; Figure 4A). Instead, surprisingly, cold stimulations induced large currents in the oocytes injected with western clawed frog TRPV3 cRNA (Figure 4A and 4B), but not in water-injected oocytes (Figure 4C). The currents induced by cold temperatures were also observed without prior heat stimulation (Figure 4B). The cold-induced currents were desensitized rapidly during the first cold stimulation and were considerably smaller during the second cold stimulation (Figure 4A). This property is different from that of mammalian TRPV3 which becomes sensitized with repeated heat stimulations [6], [8]. The average temperature threshold for activation was 16.35±0.51°C (n = 14) when analyzed with Arrhenius plots (Figure 4D).

**Figure 5:**
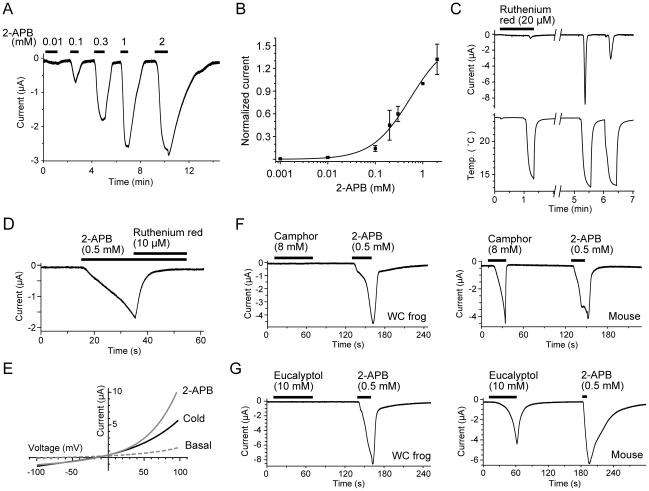
The activation and inhibition properties of the TRPV3 channel of the western clawed frog. The data in panels A-E were obtained from oocytes injected with TRPV3 cRNA of the western clawed frog. (A and B) Oocytes responded to 2-APB in a dose-dependent manner. A representative current trace evoked by 2-APB (A) and its dose-response curve (B). Currents were normalized to the values at 1 mM. The EC_50_ was 0.54±0.24 mM, and the Hill coefficient was 1.1±0.3. Error bar indicate SEMs. (C) Inhibition of cold-induced currents in the oocytes. Ruthenium red (20 µM) was applied prior to and during the first cold stimulation. The second and third cold stimuli were applied four minutes after washing out the ruthenium red. (D) A 2-APB (0.5 mM)-induced current in the oocyte was inhibited by ruthenium red (10 µM) even in the presence of 2-APB. (E) Current-voltage relationships of cold- and 2-APB-evoked responses in the oocyte. (F and G) Representative current traces in response to initial applications of camphor (8 mM) (F) or eucalyptol (10 mM) (G) with secondary applications of 2-APB (0.5 mM) in oocytes injected with TRPV3 cRNA of the western clawed frog (Left) or mouse (Right).

We next examined the pharmacological properties of western clawed frog TRPV3 currents. The oocytes expressing western clawed frog TRPV3 also responded to 2-APB, a known agonist of mammalian TRPV3 [13], [14], in a dose-dependent manner (Figure 4A, Figure 5A and 5B). In human, dog, and chicken, histidine residues at position 426 in TRPV3 are reported to be involved in 2-APB sensitivity [12]. The corresponding residue of western clawed frog TRPV3 was also histidine as reported previously [12] (Figure S3). We also confirmed that western clawed frog TRPV3 responded to 2-APB (Figure 5A and 5B). The 2-APB current tended to be sensitized when short-period stimulations (20 seconds) were repeatedly applied to the oocytes expressing western clawed frog TRPV3 (Figure S4A). This observation is similar to that of mammalian TRPV3, which showed sensitization upon heat, camphor, and 2-APB [6], [8], [9], [13]. In mouse TRPV3, a synergistic effect has been reported for temperature and 2-APB stimuli [13], [14]. Thus, temperature effects on 2-APB stimulation in TRPV3 of the western clawed frog were examined. Unexpectedly, cold stimulations suppressed 2-APB currents (Figure S4B), while heat stimulations showed potentiation effects (Figure S4C), implying that the activation mechanisms may be different between the cold and 2-APB responses for western clawed frog TRPV3.

Ruthenium red, a broad TRP channel antagonist [4], [6]–[8], inhibited cold-induced currents in a reversible manner (Figure 5C) and also inhibited 2-APB-induced currents (Figure 5D) in oocytes expressing western clawed frog TRPV3. Moreover, the currents induced by both 2-APB and cold temperatures showed an outwardly-rectifying current-voltage relationship with slightly negative reversal potentials (–12.35±3.24 mV, n = 4; and –9. 33±2.03 mV, n = 4 for cold- and 2-APB-induced currents, respectively; Figure 5E). These results indicate that TRPV3 of the western clawed frog is a nonselective cation channel with a property similar to that of mammalian TRPV3 [6]–[8], [14]. On the other hand, western clawed frog TRPV3 did not respond to camphor (8 mM), eucalyptol (10 mM; Figure 5F and 5G, Left), menthol (2 mM), vanillin (10 mM), and eugenol (2 mM) (Figure S4D–S4F), well known activators of mammalian TRPV3 (Figure 5F and 5G, Right) [9], [18], [19]. These observations suggest that while western clawed frog TRPV3 shares some electrophysiological properties with mammalian TRPV3, it also possesses distinct properties, which may be related to its opposite temperature sensitivity from mammalian TRPV3.

### Expression profile of TRPV3 mRNA in the western clawed frog

**Figure 6:**
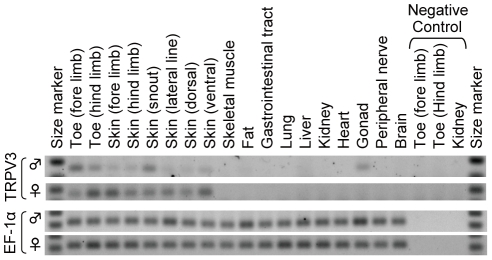
Expression of TRPV3 in the skin of the western clawed frog. Transcription profiles of TRPV3 in the western clawed frogs were examined by semi-quantitative RT-PCR. The gene for elongation factor 1α (EF-1α) was used as an internal control. For negative control experiments, RNA samples from toes of both fore- and hind-limbs as well as kidney were used. The upper and lower bands of the size markers were 200-bp and 100-bp, respectively for TRPV3, and 300-bp and 200-bp, respectively for EF-1α.

To compare the expression profiles of TRPV3 between mammals and western clawed frog, the tissue distribution of TRPV3 mRNAs in the western clawed frog was examined by semi-quantitative RT-PCR. TRPV3 mRNAs of the western clawed frog were expressed in skin from various parts of its body, toes of both fore and hind limbs, as well as testis (Figure 6). TRPV3 mRNAs were not detected in the gastrointestinal tract, peripheral nerve or brain where expression has been reported in mammals [6]–[8].

## Discussion

### Evolution of TRPV gene subfamily in vertebrates

In the present study, we performed comprehensive phylogenetic analysis on genes belonging to the TRPV subfamily from various kinds of vertebrate species (Figure 1). As previously reported [5], the *TRPV3* and *TRPV4* genes diverged earlier than the timing of the gene duplication between the *TRPV1* and *TRPV2* genes. Given that the teleost fish genes were included in the *TRPV1/2* cluster, *TRPV3* and *TRPV4* genes emerged, at the latest, in the common ancestor of teleost fishes and terrestrial vertebrates. Whether the gene duplication of *TRPV1* and *TRPV2* occurred before or after the divergence of teleost fishes and terrestrial vertebrates is unclear since statistical support for the branch connecting the terrestrial vertebrate *TRPV1* and teleost fish *TRPV1/2* genes had a moderate value. In the teleost fishes, the *TRPV1/2* genes showed copy number variation. Only one copy of the *TRPV1/2* genes was found for stickleback and zebrafish, while two copies were found for medaka, torafugu, and spotted green pufferfish (Figure 1 and Figure 2A). Since teleost fish *TRPV1/2* genes were clustered together with 99% bootstrap value, the gene duplication events producing teleost fish TRPV1/2s and vertebrate TRPV1 and TRPV2 must have independently occurred in each lineage. Around the genomic region encompassing the two paralogous *TRPV1/2* genes, syntenic relationships are preserved (Figure 2A), suggesting that the two copies of the *TRPV1/2* genes were produced by the whole genome duplication that occurred in the common ancestor of teleost fishes [20]. One copy has subsequently been lost in the stickleback and zebrafish lineages. In contrast, given that the *TRPV1-TRPV3* genes are closely located in the terrestrial vertebrate genome, they were produced by tandem gene duplications.

In the course of phylogenetic analysis, we found four novel *TRPV* genes that had yet to be reported (Figure 1). Three of these novel genes clustered with the *TRPV5* and *TRPV6* genes - two genes were from platypus (*TRPV8* and *TRPV9*) and one from the western clawed frog (*TRPV8*). That these three genes split from the *TRPV5/6* cluster suggests that they emerged in the common ancestor of teleost fish and terrestrial vertebrates. We also found another novel *TRPV* gene (*TRPV7*) in platypus that formed a monophyletic cluster with the *TRPV1-TRPV4* clusters (Figure 1). Since *TRPV7* was located outside of the vertebrate *TRPV1-TRPV4* genes in the phylogenetic tree, we expected that other vertebrate species also possess orthologous genes. Our search for the orthologs to platypus *TRPV7* in several vertebrate genome sequences, however, failed to find any orthologous genes. One of the explanations for this observation is that the *TRPV7* gene may have emerged in the common ancestor of teleost fish and terrestrial vertebrates, and has been lost in most of the lineages. However, this scenario is unlikely because we have to assume independent gene loss events in the lineages leading to the different vertebrate classes. Another explanation is that *TRPV7* was specifically produced in the lineage leading to platypus and a large amount of amino acid substitutions have subsequently been accumulated. The fact that *TRPV7* was located between the *TRPV1* and *TRPV3* genes in the genome sequence of platypus suggests that *TRPV7* was produced from either *TRPV1* or *TRPV3*, or from both genes (Figure 2B). At present, it is unclear if platypus *TRPV7* is a functional gene; further characterization of *TRPV7* will prove interesting for future study since phylogenetically it is closely related to the *TRPV1-TRPV4* genes that code for temperature sensitive channels.

In dog, cow, and horse, we found one copy each of the *TRPV1-TRPV6* genes, as has been found for human and rodents (Figure 1). Chicken and green anole possessed one copy each of the *TRPV1-4* genes. Western clawed frog possessed one copy each of the *TRPV1-3* genes and possessed six copies of *TRPV4* genes as reported previously [5]. Copy numbers also varied for *TRPV1/2* genes among teleost fishes and for *TRPV5-9* genes among vertebrate species. In addition, *TRPV3* has been lost in the teleost fishes (Figure 1 and Figure 2B). In conclusion, the repertoires of the *TRPV* gene subfamily in vertebrates are essentially conserved, but gene duplication and loss events that occurred in specific lineages resulted in copy number variation; which potentially contributed to adaptation in the respective species.

### Functional evolution of the TRPV3 channel in vertebrates

In the present study, we attempted to identify thermoTRPs that have changed their functional properties within specific evolutionary lineages as these thermoTRPs must have been involved in adaptation to thermal environments. In some cases, the functional shift of thermoTRPs was accompanied by diversifications of amino acid sequences. To identify these changes, we performed detailed comparative analyses of mammalian thermoTRP homologs utilizing the genome sequence database of various vertebrate species. In the first phase of our study, we comprehensively collected thermoTRP homologs from various vertebrate species and conducted comparative analyses (Figure 1 and Figure 2) [5]. These analyses showed that the N- and C-terminal regions of the western clawed frog were missing from the predicted gene in the genome sequence database. A search for the homologous sequences to mammalian TRPV3 terminal regions in the genome sequence database of the western clawed frog failed to detect such regions. Thus we predicted that both terminal regions of western clawed frog TRPV3 are different those of from mammalian orthologs. To elucidate the amino acid sequences of western clawed frog TRPV3, the cDNA sequence was determined and the deduced amino acid sequence was compared to those of other vertebrate TRPV3s. As expected, the N- and C-terminal regions of TRPV3 in the western clawed frog were highly diversified from those regions of TRPV3 in other terrestrial vertebrate species, although the central portions were relatively conserved among all terrestrial vertebrates examined (Figure 3B, 3C, and Figure S3). Characterization of western clawed frog TRPV3 channel properties revealed striking differences from those of mammalian TRPV3 channels. The TRPV3 channel of the western clawed frog was not activated by chemical compounds that are known to activate the mammalian TRPV3 channel (Figure 5F and 5G) [9], [18], [19]. Furthermore, the TRPV3 channel of the western clawed frog was activated by cold temperatures whereas the mammalian TRPV3 channels has been reported to be activated by warm temperatures (Figure 4) [6]–[8]. Thus through a combination of interdisciplinary approaches including bioinformatics, molecular evolution, molecular biology, and electrophysiology, we were able to successfully identify a thermoTRP that has undergone a functional shift during the course of vertebrate evolution.

Opposite temperature sensitivities among orthologs have been reported in other thermoTRPs. For instance, the TRPA1 channels are activated by warm temperatures in several snake and insect species [21], [22], while it is activated by cold temperatures in mouse, although cold activation of mouse TRPA1 is the subject of some debate [23]–[27]. In the present study, we clearly demonstrate that the *TRPV3* gene of the western clawed frog and mammals are orthologous genes by showing a monophyletic relationship among them (Figure 1) as well as conserved syntenic relationships of the genes flanking the *TRPV3* genes among terrestrial vertebrate species (Figure 2B). These results indicate that TRPV3 channels have acquired opposite temperature sensitivities during the course of terrestrial vertebrate evolution. This, in turn, indicates that the temperature sensitivity of thermoTRPs is not always stable but can dynamically change, even reveres in some cases, during the course of evolution.

The molecular determinants for the difference in temperature sensitivities are not clear at present, but several lines of evidence suggest the N- and C-terminal regions as candidate domains. First, amino acid sequences of TRPV3 channels in these regions are highly divergent between mammals and the western clawed frog (Figure 3B, 3C, and Figure S3). Second, the C-terminal regions of thermoTRP channels have been reported to be involved in the modulations of temperature sensitivities. It has been shown that the swapping of the C-terminal regions between TRPV1 and TRPM8 channels of rat results in an exchange of temperature sensitivities [28]. Furthermore, it has also been reported that gradual truncations of the C-terminal regions of rat TRPV1 channels gradually shift temperature thresholds for activation [29]. Additionally, in the case of TRPV2, the N- and C-terminal regions are reported to play crucial roles in heat sensitivity in rodents [30]. Lastly, a chimeric mutant of the TRPV3 channel (in which the N- and C-terminal regions of mouse TRPV3 were fused to the central portion of TRPV3 from the western clawed frog) exhibited warm temperature activation when expressed in cultured mammalian cells (HEK293T) [11]. This chimeric mutant was also reported to be activated by camphor, thus the N- and C-terminal regions are likely to be involved in both chemical and temperature sensitivity of the TRPV3 channel. Future detailed study using chimeric mutants of the TRPV3 channel will be necessary to understand the molecular basis for the differences in temperature as well as chemical sensitivities of TRPV3 channels between mammals and the western clawed frog.

What is the physiological role of TRPV3 in the western clawed frog? TRPV3 was mainly expressed in the skin in the western clawed frog similar to the expression pattern of mammalian TRPV3 (Figure 6). In mammals, it has been proposed that thermal stimuli perceived by the skin are transmitted to peripheral nerves [3], [4], [31], [32]. Therefore, TRPV3 of the western clawed frog is likely to be involved in sensing temperatures at the body surface. The western clawed frog is a fully aquatic anuran that inhabits tropical areas, and its optimal ambient temperature range is 22–28°C [33], [34]. Temperatures below 18–20°C have detrimental effects [35], [36], and here we show that the temperature threshold for activation of the TRPV3 channel is slightly below its temperature limit (about 16°C) (Figure 4D). Thus, the physiological role of the TRPV3 channel is likely to detect noxious cold temperatures in the western clawed frog.

It is expected that the physiological importance of warm temperature perception is higher in homeothermic vertebrates than for ectothermic vertebrates. TRPV3 channels serve crucial roles in homeothermic mammals by detecting innocuous temperatures near the body temperatures such as in mouse [9], while it serves as a sensor to detect noxious cold temperatures in ectothermic vertebrates such as the western clawed frog (Figure 4). Similar observations have been reported for TRPM8 channels that act as cold temperature receptors. Activation temperatures of TRPM8 channels of the western and African clawed frog are much lower than those of rat and chicken TRPM8 channels [37]. Since body temperatures of frogs are lower than mammals and birds, it has been interpreted that the shift in temperature sensitivity of TRPM8 channels reflects the differences in the physiological requirements of body temperatures between homeothermic and ectothermic vertebrates. Characterization of TRPV3 channels from more diverse amniote species including both homeothermic and ectothermic animals will provide new insights into the functional evolution of temperature receptors related to homeothermy in vertebrates.

**Figure 7:**
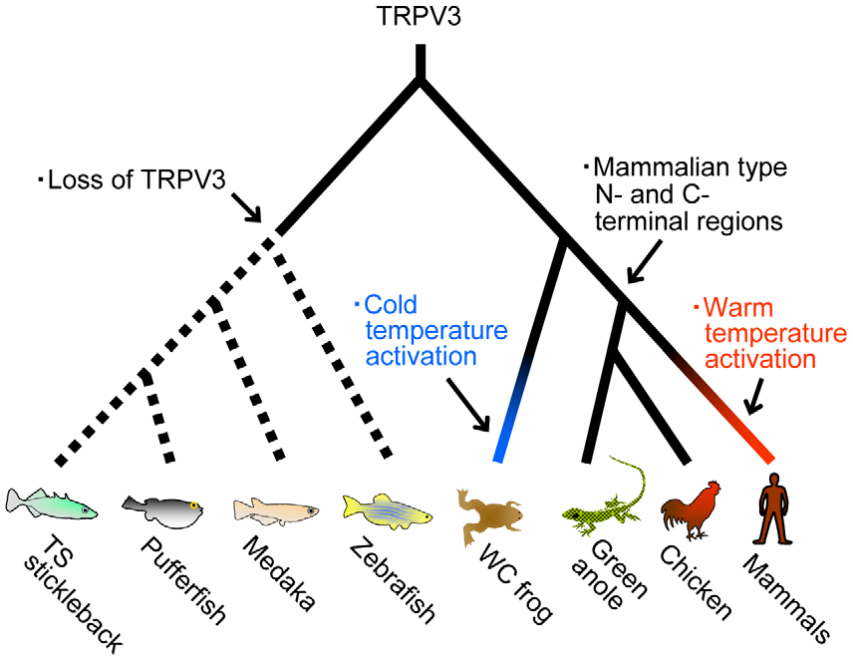
The evolutionary changes of TRPV3 channels in the vertebrate lineages. The major evolutionary events are indicated on the respective branches. The amino acid sequences of the N- and C-terminal regions of the TRPV3 channels were conserved among amniote species, while the N- and C-terminal regions of TRPV3 in the western clawed frog were highly diversified from those regions of TRPV3 in other terrestrial vertebrate species. The ancestral states of the terminal regions are ambiguous since teleost fishes have lost the *TRPV3* gene. Western clawed frog and mammals acquired opposite temperature sensitivities of TRPV3 channels; however, the timing of the shift is not clearly determined since the temperature sensitivities of TRPV3 channels of birds and reptiles have not been reported.

In conclusion, detailed comparative analyses on the *TRPV* subfamily performed in the present study identified novel *TRPV* genes that have not been reported previously (Figure 1) and also elucidated the flexible nature of *TRPV3* in vertebrate evolution (Figure 7). The *TRPV3* gene emerged in the common ancestor of teleost fishes and terrestrial vertebrates but has subsequently been lost in teleost fish lineages. Terrestrial vertebrates retained the TRPV3 channel, however, the western clawed frog and mammals acquired opposite temperature sensitivity to detect environmental temperatures suitable for their respective species. Thus the results of the present study reveal that thermoTRPs can dynamically change channel properties to adapt to different thermal environments during the course of evolution.

## Materials and Methods

### Retrieving nucleotide sequences of the *TRPV* genes

The *TRPV1-5* genes from human, mouse, rat, chicken, western clawed frog, zebrafish, and torafugu were previously collected [5]. For the present study we collected *TRPV* homologous genes from several mammalian species, green anole, western clawed frog, and teleost fishes utilizing the orthologue prediction based on the draft genome sequence database published by Ensembl (http://www.ensembl.org/index.html).

### Molecular phylogenetic analysis

Multiple sequence alignments were performed using the CLUSTAL W algorithm [38], with minor manual adjustments. Evolutionary distances between the amino acid sequences were calculated using the central conserved portions containing the ankyrin repeat and transmembrane domains (381 residues) by applying the JTT model [16] after all alignment gap sites were eliminated. The phylogenetic tree was then reconstructed using the minimum-evolution method [17]. The statistical confidence of each branch in the phylogenetic tree was estimated by the bootstrap method with 1,000 replications [15]. All of the above analyses were performed using MEGA4 software [39]. The *TRPV* genes and species used for phylogenetic reconstruction are listed in Table S1.

### Western clawed frog

All procedures involving the care and use of animals were approved by the National Institute for Physiological Sciences. Western clawed frogs (*Xenopus tropicalis*) were kindly provided by the National Bio-resource Project (NBRP) of the Ministry of Education, Science, Sports and Culture of Japan. The western clawed frog strain used was the Yasuda line [34].

### Sequencing and cloning of the *TRPV3* gene of the western clawed frog

Using total RNA extracted from the toe of a fore-limb of an adult female western clawed frog as the template, a cDNA fragment spanning the 5′- to 3′-UTR of the *TRPV3* gene was amplified by RT-PCR and 5′- to 3′-RACE. A DNA fragment containing the western clawed frog *TRPV3* gene was amplified by RT-PCR and cloned into the pGEMHE vector. The PCR primers used are listed in Table S2. The mouse *TRPV3* gene that was cloned into the pcDNA3 vector (Invitrogen) was the kind gift of Mike Caterina (Johns Hopkins, Baltimore, USA) and was subcloned into pOX+ vector.

### Oocyte electrophysiology

The TRPV3 channel of the western clawed frog was heterologously expressed in oocytes of the African clawed frog *Xenopus laevis*, and ionic currents were recorded using the two-electrode voltage-clamp method. Western clawed frog TRPV3 cRNA was injected into defolliculated oocytes and ionic currents were recorded 1–4 days post-injection. The oocytes were voltage-clamped at −60 mV. All chemical compounds were diluted into ND96 bath solution and applied to the oocytes by perfusion. For thermal stimulations, heated or cold ND96 bath solutions were applied by perfusion. The current-voltage relationship was obtained using 200 ms voltage-ramp pluses from −100 to +100 mV applied every 1.5 seconds. The data values are expressed as mean ± SEM.

### Semi-quantitative RT-PCR

Total RNA was extracted from skin from various parts of the body, toes of the fore- and hind-limbs, thigh skeletal muscle, fat body, gastrointestinal tract, lung, liver, kidney, heart, testis, ovary, peripheral nerve, and brain of male and female adult western clawed frogs. A 149-bp and a 206-bp cDNA fragment containing the TRPV3 and EF-1α (as internal control) genes, respectively, were amplified by RT-PCR.

Details of the Materials and Methods are described in Text S1.

## Supporting Information

Figure S1 The nucleotide sequence of TRPV3 cDNA of the western clawed frog. The boundaries of the exons are shown with the lengths of the introns (bp) that are located between the exons. Note that intron length between exons 2 and 3 is not clearly determined since the nucleotide sequence information is partially lacking in this intron. The locations of the primers used in the present study for the forward and reverse directions are delineated by blue and orange boxes, respectively. The deduced amino acid sequence is shown below the cDNA nucleotide sequence.
(TIF)

Figure S2 The nucleotide sequence of the genomic region containing exon 2 in the *TRPV3* gene of the western clawed frog. The nucleotide sequence of exon 2 is shadowed. The locations of the primers are indicated by lines with arrows to indicate direction.
(TIF)

Figure S3 The amino acid alignment of the TRPV3 channels of terrestrial vertebrates. The amino acid identical to, similar to, and different from consensus residues are indicated by red, blue, and black letters, respectively. Bold and thin lines delineate putative ankyrin (ANK) repeat and transmembrane domains (TM), respectively. Histidine residue involved in 2-APB sensitivity is marked with a dot. The locations of the transmembrane domains of the human TRPV3 channel are from Smith et al. [7]. The locations of the transmembrane domains of the western clawed frog were predicted by TMpred (http://www.ch.embnet.org/software/TMPRED_form.html). Note that the amino acid residues of the green anole TRPV3 channel are partially missing (indicated by ‘X’) due to incompleteness of the genome sequence database.
(TIF)

Figure S4 The activation properties of the TRPV3 channel of the western clawed frog. All the data were obtained from oocytes injected with TRPV3 cRNA of the western clawed frog. (A) The responses of the oocytes upon repeated 2-APB stimulations. 2-APB (0.5 mM) was applied repeatedly in a short period (20 seconds). Cold (B) or warm (C) temperature effects on 2-APB currents. Cold or warm stimulations were applied to the oocytes during the 2-APB (0.2 mM) administration. (D-F) Representative current traces in responses to initial applications of menthol (2 mM) (D), vanillin (10 mM) (E), or eugenol (2 mM) (F) with secondary applications of 2-APB (0.5 mM) in the oocytes.
(TIF)

Table S1 Summary table listing information for the genes used in the present study. The names of the species and genes are listed with their gene identifiers from the databases.
(TIF)

Table S2 List of the primers used in the present study. Sequence, Tm, and Location for each of the primers are listed.
(TIF)

Text S1 Materials and Methods are described in detail.
(DOC)
