# Supplementary figures and images for: Evolution of Vertebrate Transient Receptor Potential Vanilloid 3 Channels: Opposite Temperature Sensitivity between Mammals and Western Clawed Frogs
Source: PLoS Genet. 2011 Apr 7;7(4):e1002041. doi: 10.1371/journal.pgen.1002041 (PMC3072374; doi:10.1371/journal.pgen.1002041)

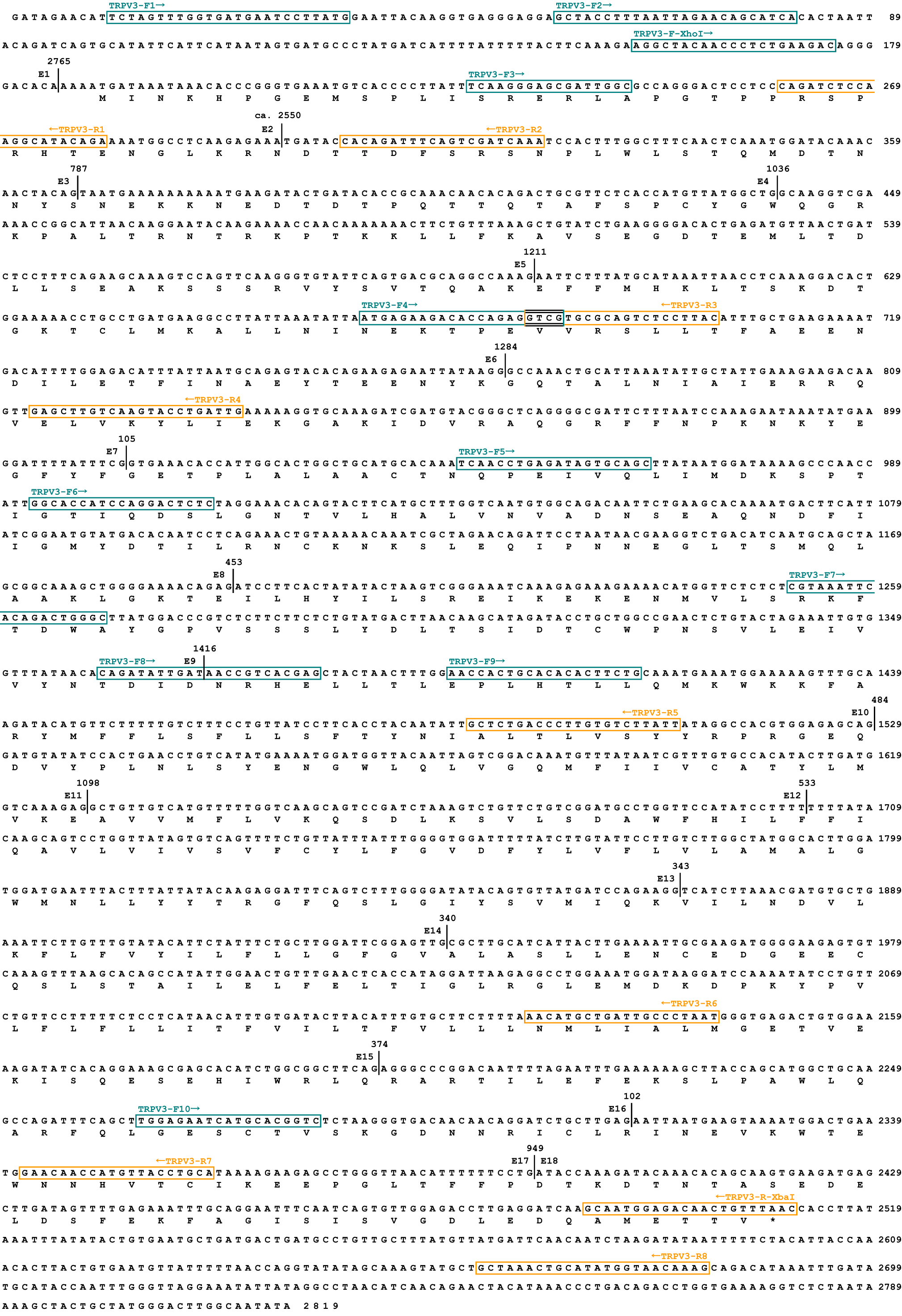

Supplement: Figure S1 — The nucleotide sequence of TRPV3 cDNA of the western clawed frog. The boundaries of the exons are shown with the lengths of the introns (bp) that are located between the exons. Note that intron length between exons 2 and 3 is not clearly determined since the nucleotide sequence information is partially lacking in this intron. The locations of the primers used in the present study for the forward and reverse directions are delineated by blue and orange boxes, respectively. The deduced amino acid sequence is shown below the cDNA nucleotide sequence. (TIF) [file pgen.1002041.s001.tif]

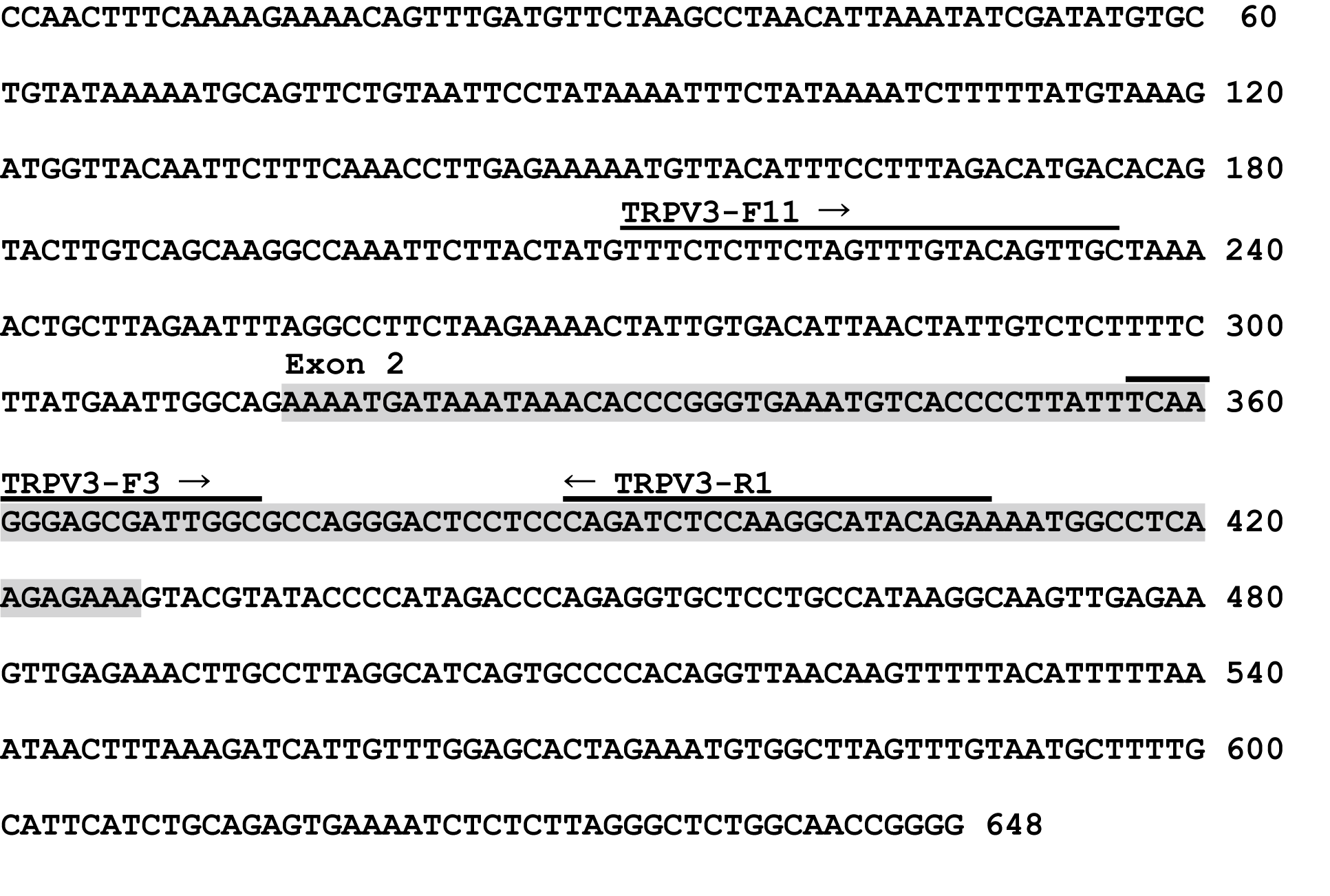

Supplement: Figure S2 — The nucleotide sequence of the genomic region containing exon 2 in the TRPV3 gene of the western clawed frog. The nucleotide sequence of exon 2 is shadowed. The locations of the primers are indicated by lines with arrows to indicate direction. (TIF) [file pgen.1002041.s002.tif]

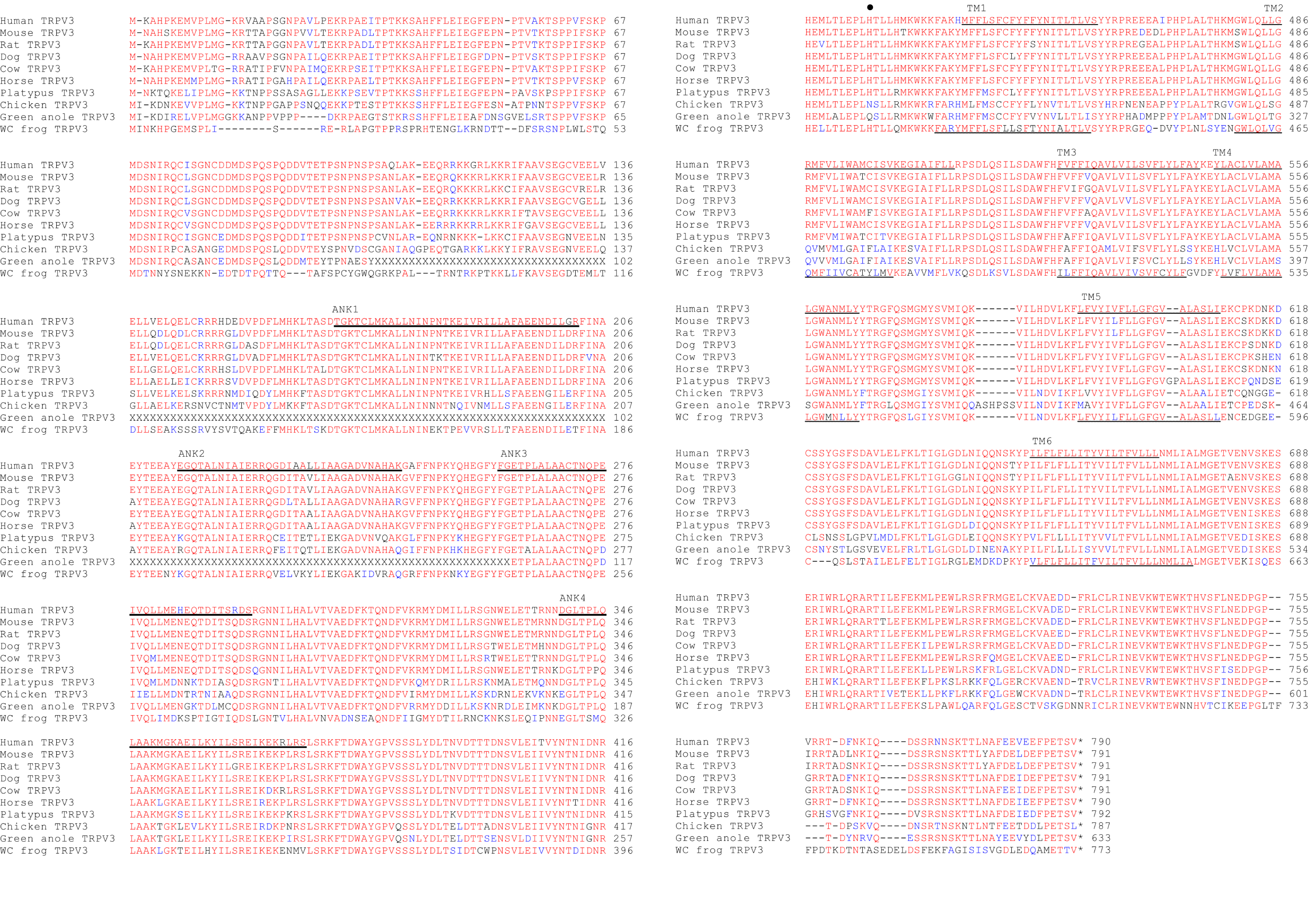

Supplement: Figure S3 — The amino acid alignment of the TRPV3 channels of terrestrial vertebrates. The amino acid identical to, similar to, and different from consensus residues are indicated by red, blue, and black letters, respectively. Bold and thin lines delineate putative ankyrin (ANK) repeat and transmembrane domains (TM), respectively. Histidine residue involved in 2-APB sensitivity is marked with a dot. The locations of the transmembrane domains of the human TRPV3 channel are from Smith et al. [7]. The locations of the transmembrane domains of the western clawed frog were predicted by TMpred (http://www.ch.embnet.org/software/TMPRED_form.html). Note that the amino acid residues of the green anole TRPV3 channel are partially missing (indicated by ‘X’) due to incompleteness of the genome sequence database. (TIF) [file pgen.1002041.s003.tif]

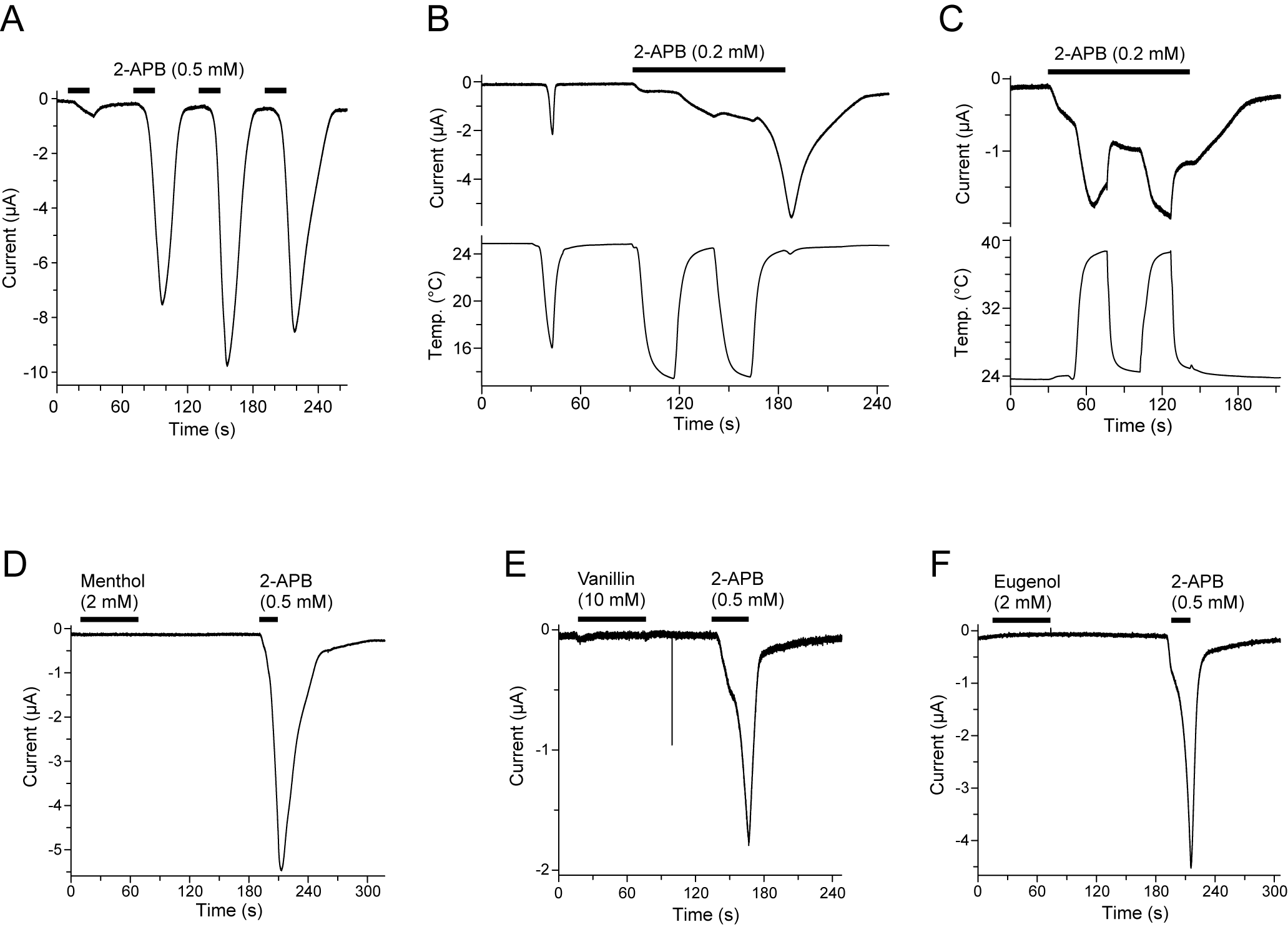

Supplement: Figure S4 — The activation properties of the TRPV3 channel of the western clawed frog. All the data were obtained from oocytes injected with TRPV3 cRNA of the western clawed frog. (A) The responses of the oocytes upon repeated 2-APB stimulations. 2-APB (0.5 mM) was applied repeatedly in a short period (20 seconds). Cold (B) or warm (C) temperature effects on 2-APB currents. Cold or warm stimulations were applied to the oocytes during the 2-APB (0.2 mM) administration. (D-F) Representative current traces in responses to initial applications of menthol (2 mM) (D), vanillin (10 mM) (E), or eugenol (2 mM) (F) with secondary applications of 2-APB (0.5 mM) in the oocytes. (TIF) [file pgen.1002041.s004.tif]

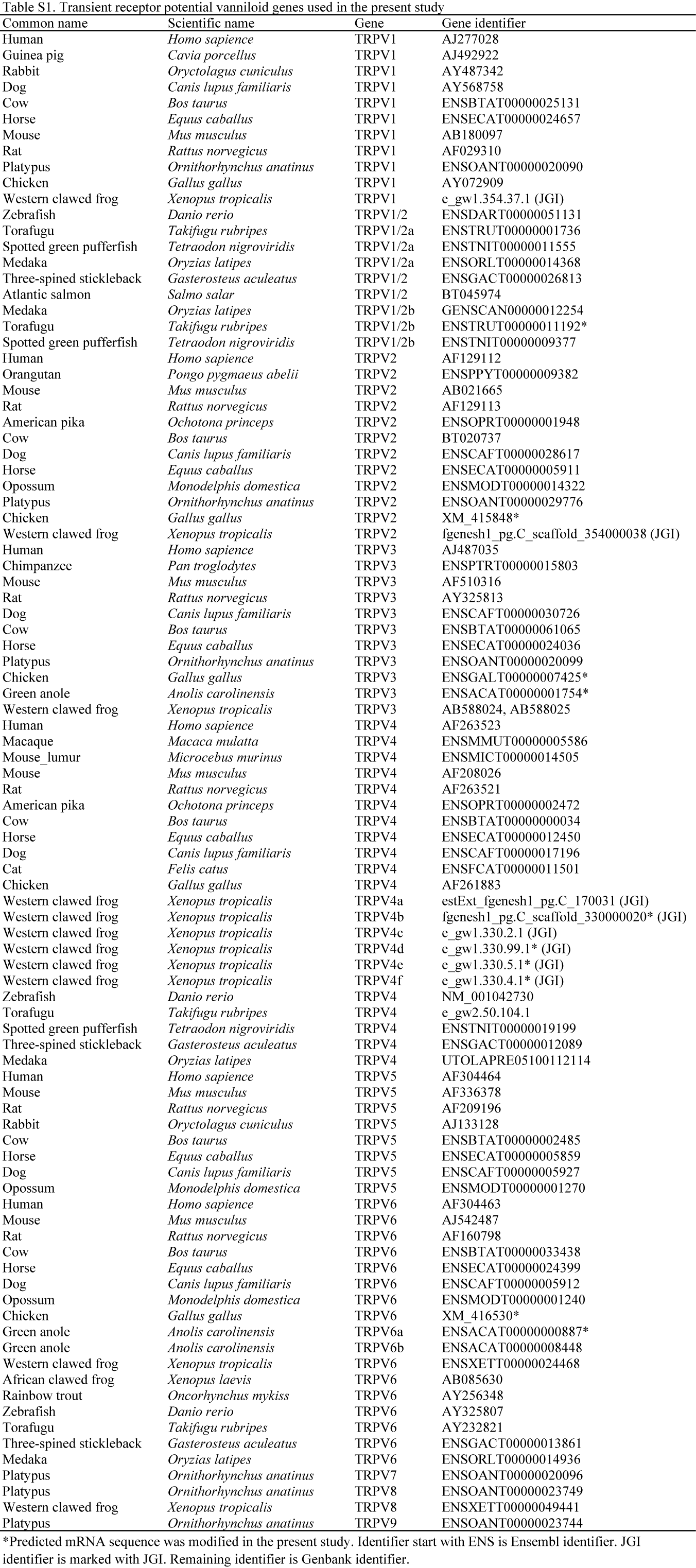

Supplement: Table S1 — Summary table listing information for the genes used in the present study. The names of the species and genes are listed with their gene identifiers from the databases. (TIF) [file pgen.1002041.s005.tif]

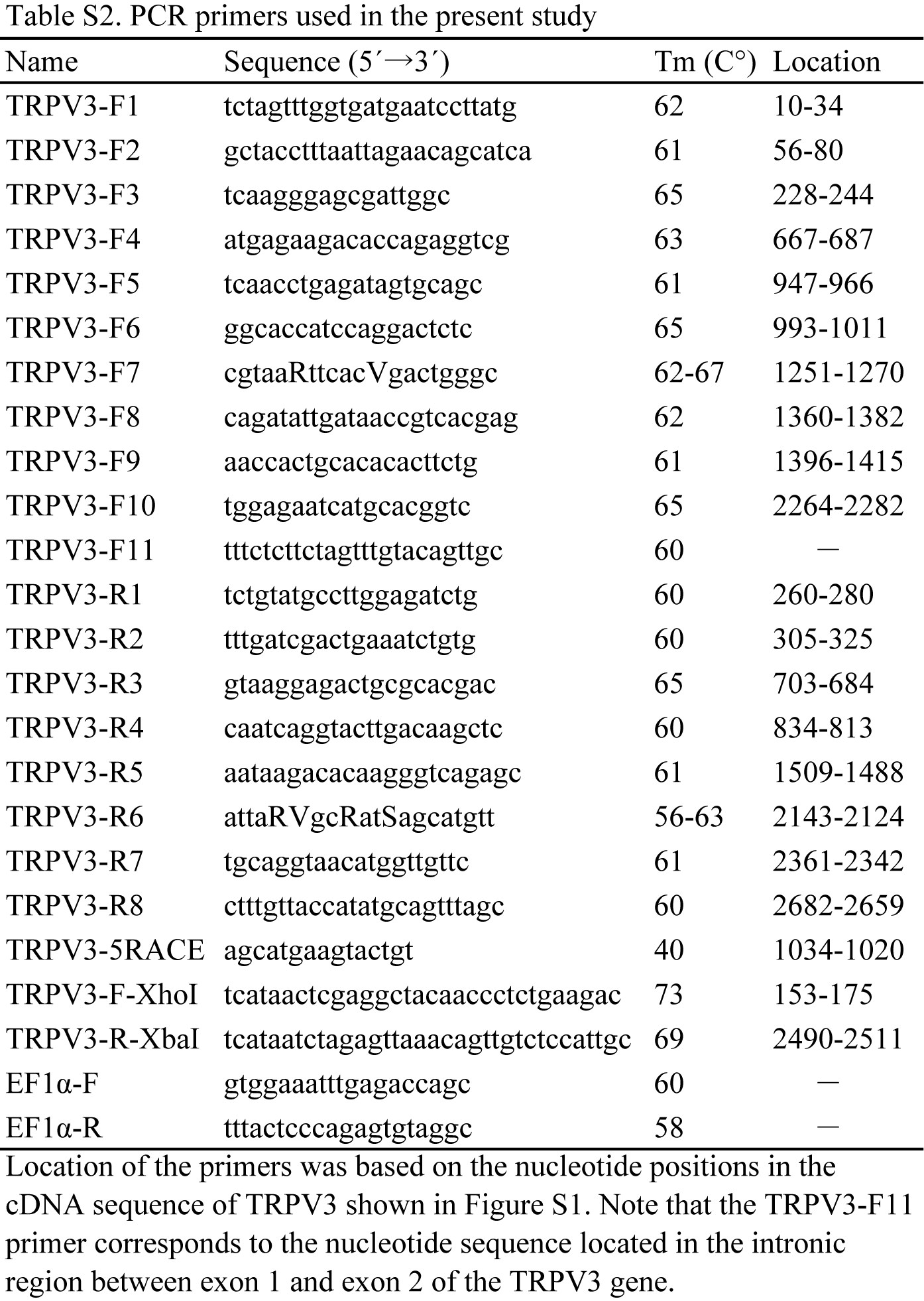

Supplement: Table S2 — List of the primers used in the present study. Sequence, Tm, and Location for each of the primers are listed. (TIF) [file pgen.1002041.s006.tif]
