# Supplementary material for: Evolution of Vertebrate Transient Receptor Potential Vanilloid 3 Channels: Opposite Temperature Sensitivity between Mammals and Western Clawed Frogs
Source: PLoS Genet. 2011 Apr 7;7(4):e1002041. doi: 10.1371/journal.pgen.1002041 (PMC3072374; doi:10.1371/journal.pgen.1002041)
Supplement: Text S1 — Materials and Methods are described in detail. (DOC) [file pgen.1002041.s007.doc]

**Supporting Information**

**Evolution of Vertebrate Transient Receptor Potential Vanilloid 3 Channels: Opposite Temperature Sensitivity between Mammals and Western Clawed Frogs**

Shigeru Saito, Naomi Fukuta, Ryuzo Shingai, and Makoto Tominaga

**Supporting Materials and Methods**

Retrieving nucleotide sequences of the TRPV genes

*TRPV1-5* genes from human, mouse, rat, chicken, western clawed frog, zebrafish, and torafugu were previously collected [1]. The *TRPV* genes collected for the present study were searched utilizing the orthologue predictions based on the draft genome sequence database published by Ensembl (http://www.ensembl.org/index.html). Some of the *TRPV* genes have portions that have not been sequenced yet, and these *TRPV* genes were not suitable for phylogenetic reconstruction due to the reduction in the numbers of the amino acid residues analyzed. Thus, only the *TRPV* genes whose conserved central region (from the first ankyrin repeat domain to the TRP domain) has been sequenced were retrieved from the draft genome sequence databases. The *TRPV* genes and species used in the present study are listed in Table S1. TBLASTN searches were performed on the respective draft genome sequences when necessary. Some of the *TRPV* genes collected in the present study have portions that are likely misannotated in the Ensembl database. We have modified the annotations according to the procedures previously described [1]. Orthologous/paralogous relationships of collected *TRPV* genes were confirmed by phylogenetic analysis and confirmation of syntenic relationships. To detect conserved syntenic relationships in the genomic region encompassing the *TRPV1-TRPV3* genes, the gene orders around them were compared among several vertebrate species using Ensembl database such as location-based displays and orthologue predictions.

Sequencing and cloning of the *TRPV3* gene of the western clawed frog

Total RNA was extracted from the toe of a fore-limb excised from an adult female western clawed frog. The excised tissue was placed in a 1.5 ml tube and frozen with liquid nitrogen. Total RNA was extracted using TRIZOL LS reagent (Life technologies) according to the manufacturer’s instructions. Extracted total RNA was resuspended in 50 µl of RNase free water.

RT-PCR was performed to obtain the DNA fragment containing TRPV3 cDNA. RT-PCR and 3′-RACE were performed using ReverTra -Plus- kit (Toyobo). In a typical reaction, reverse transcription was carried out in 20 µl of reaction mixture containing 4 µl of 5 × RT buffer, 2mM each dNTP, 40 units of RNase Inhibitor, 100 units of ReverTra Ace, 500 ng of total RNA extracted from the respective tissue of western clawed frog, and 40 pmol of a primer. The mixture was incubated for 60 min at 42°C and then for 5 min at 85°C. PCR was performed in 25 µl of reaction mixture containing 2.5 µl of 10 × PCR buffer, 1.5 mM MgSO_4_, 0.2 mM of each dNTP, 0.5 units of KOD –Plus– polymerase (Toyobo), 10 pmol of each forward and reverse primer, and 0.5 µl of the reverse transcription product. The cycling conditions were according to the supplier’s instructions. The primers used in the present study are listed in Table S2.

To obtain the cDNA fragment containing the *TRPV3* gene, reverse transcription was performed using total RNA extracted from the toe of a fore-limb of a female western clawed frog and the GeneRacer Oligo dT primer (Invitrogen). Using this reverse transcription product as template DNA, RT-PCR was performed with the TRPV3-F4 and TRPV3-R5 primers that amplified the central conserved region of the *TRPV3* gene. An approximately 850 bp DNA fragment was successfully amplified and directly sequenced with the same primers. To obtain the cDNA fragment containing the 3′ end of the *TRPV3* gene, reverse transcription was performed using total RNA extracted from the toe of a fore-limb of a female western clawed frog and Gene-Racer Oligo dT primer (Invitrogen). Using this reverse transcription product as template DNA, the first PCR was carried out with the frog TRPV3-F4 and GeneRacer 3′ primer (Invitrogen), followed by the second PCR with the frog TRPV3-F7 and Gene Racer 3′ nested primer (Invitrogen). The amplified DNA fragment was sequenced with the frog TRPV3-F9, frog TRPV3-F10, frog TRPV3-R5, frog TRPV3-R6, frog TRPV3-R7, and frog TRPV3-R8 primers. To obtain the 5′ end of TRPV3 cDNA, 5′-RACE was carried out using the 5′-Full RACE Core Set (TAKARA) according to the supplier’s instructions. Reverse transcription was performed using 1.3 µg of total RNA extracted from the toe of a fore-limb of a female western clawed frog and the frog TRPV3-5RACE primer. The first PCR was performed with the frog TRPV3-F5 and frog TRPV3-R4 primers, followed by the second PCR with the frog TRPV3-F6 and frog TRPV3-R3 primers. The amplified DNA fragment was sequenced using frog TRPV3-F6 and frog TRPV3-R3 primers.

The comparison of the cDNA sequence determined in the present study with the genome sequence database of the western clawed frog revealed that the nucleotide sequence corresponding to the second exons (114 bp) did not exist in the database. To confirm the result obtained by 5′-RACE, the cDNA fragment from exons 1 to 6 was amplified by RT-PCR. PCR primers (frog TRPV3-F1 and TRPV3-F2) that correspond to the sequences near the 5′ end of the TRPV3 cDNA were designed and RT-PCR was conducted. Reverse transcription was performed using total RNA extracted from the toe of a fore-limb of a female western clawed frog and the GeneRacer Oligo dT primer (Invitrogen). Using this reverse transcription product as template DNA, the first PCR was performed with the frog TRPV3-F1 and frog TRPV3-R4 primers, followed by the second PCR with the frog TRPV3-F2 and frog TRPV3-R3 primers. The amplified DNA fragment was sequenced with the same primers. An approximately 650-bp DNA fragment that contained the second exon was obtained.

To further confirmed the existence of the second exon, the genomic region of the *TRPV3* gene was amplified and sequenced. Total DNA was extracted from the liver of an adult female western clawed frog according to the method of Boom *et al*. [2]. The DNA fragment containing the genomic region from exons 1 to 3 was amplified with the frog TRPV3-F1 and frog TRPV3-R2. Using this PCR product as template DNA, the DNA fragments containing the genomic region from exons 1 to 2 and from exons 2 to 3 were amplified with the primer pairs frog TRPV3-F1 and frog TRPV3-R1, and frog TRPV3-F3 and frog TRPV3-R2, respectively. The amplified DNA fragment was sequenced with frog TRPV3-F1, frog TRPV3-F3, frog TRPV3-F11, frog TRPV3-R1, and frog TRPV3-R2. The second exon was located in the genomic portions that had yet to be sequenced in the genome sequence project (Figure S1B). Therefore, the existence of the second exon was not an artifact.

For cloning of the TRPV3 gene of the western clawed frog, DNA fragment containing the full length coding region of TRPV3 was amplified by RT-PCR with the frog TRPV3-F-XhoI and frog TRPV3-R-XbaI primers. The amplified DNA fragment was first cloned into pcDNA3, then it was subcloned into the pGEMHE vector suitable for the *Xenopus* oocyte expression system. The PCR primers used are listed in Table S2. The mouse *TRPV3* gene that was cloned into the pcDNA3 vector (Invitrogen) was the kind gift of Mike Caterina (Johns Hopkins, Baltimore, USA) and was subcloned into oocyte expression vector pOX+.

Oocyte electrophysiology

The ovarian lobes were excised from mature female *Xenopus laevis*. In a typical experiment, excised ovarian lobes were incubated in MBSH-PS buffer (88 mM NaCl, 1 mM KCl, 2.4 mM NaHCO_3_, 0.82 mM MgSO_4_, 0.33 mM Ca(NO_3_)_2_, 0.41 mM CaC1_2_, 15 mM

HEPES (pH 7.4), and 10 μg/mL of each streptomycin sulfate and benzylpenicillin potassium) containing 2.5 mg/ml collagenase A (Roche) with mild rotation for 2 – 3 hours at a room temperature to remove the follicle membrane. This step was repeated once. Healthy looking oocytes (stages V – VI) were selected and incubated at 17°C over night in MBSH-PS buffer. Next, approximately 10 – 25 ng of western clawed frog TRPV3 cRNA was injected into oocytes and incubated at 17°C. The cRNA injected oocytes were used for electrophysiological recordings 1-4 days post-injection.

The two-electrode voltage-clamp recording method was used to measure the ionic current of the oocytes. Electrodes were pulled from borosilicate glass using a flaming/brown pipette puller P-97 (Sutter Instrument). Electrodes were filled with 3M KCl and resistances were adjusted to 0.3 – 1.5 MΩ. The oocytes were voltage-clamped at – 60 mV and currents were recorded with an OC-725C amplifier (Warner Instrument) and digitized at 10 kHz by digidata 1440 (Axon Instrument). All chemical compounds were diluted into ND96 bath solution (96 mM NaCl, 2 mM KCl, 1.8 mM CaC1_2_, 1mM MgC1_2_, and 5 mM pH 7.4 HEPES) and applied by perfusion. Camphor (3 M) was dissolved in ethanol for the stock solution. Menthol (2 M), vanillin (3.8 M), and eugenol (2 M) were dissolved in dimethyl sulfoxide for stock solutions. Stock solution for ruthenium red (10 mM) was dissolved in water. All the stock solutions were diluted into ND96 bath solution just before use. Eucalyptol was dissolved directly into the ND96 bath solution. For the heat and cold stimulations, heated or cooled ND96 bath solutions were applied by perfusion and temperatures were recorded with a TC-344B (Warner Instrument) by placing the thermistor just beside the oocytes. The current-voltage relationship was obtained using 200 ms voltage ramp from -100 to +100 mV generated once per 1.5 seconds.

Semi-quantitative RT-PCR

Skin from various parts of the body, toes of the fore- and hind-limbs, thigh skeletal muscle, fat body, gastrointestinal tract, lung, liver, kidney, heart, testis, ovary, peripheral nerve, and brain were dissected from male and female adult western clawed frogs. Total RNA was extracted using TRIZOL LS reagent (Life technologies). To remove the contaminated genomic DNA, total RNA was purified with RNeasy Plus Mini kit (Qiagen). Reverse transcription was performed in 20 µl of reaction mixture containing 4 µl of 5 × RT buffer, 2mM each dNTP, 40 units of RNase Inhibitor, 100 units of ReverTra Ace, 500 ng of purified RNA, and 50 pmol of a Origo dT(20) primer using ReverTra Ace qPCR RT kit (Toyobo). The PCR was performed in 25µl of reaction mixture using GoTaq Green Master Mix (Promega). As an internal control, elongation factor 1α (EF-1α) cDNA was amplified using the EF1α-F and EF1α-R primers. These two primers are specific to sequences of exons three and four of the western clawed frog EF-1α gene and produce a 206-bp DNA fragment. The reaction condition was 95°C for 2 min, 23 cycles of 95°C for 30 sec, 58°C for 30 sec, and 72°C for 30 sec. The amplified DNA fragment was identified as the EF-1α by direct sequencing. For amplification of TRPV3 cDNA, the frog TRPV3-F8 and frog TRPV3-R5 primers were used. The frog TRPV3-F8 primer corresponds to the sequence located at the boundary of exons 9 and 10, and the frog TRPV3F-R5 primer at exon 10. This primer pair amplifies a 149 bp DNA fragment. The amplified DNA fragment was identified as the *TRPV3* sequence by direct sequencing. The typical reaction condition is the same as that of EF-1α except that the cycles were repeated 37 times. For the above mentioned PCR cycle conditions, we confirmed that the RT-PCR was within a linear range of amplification.

**Supporting References**

1. Saito S, Shingai R (2006) Evolution of thermoTRP ion channel homologs in vertebrates. Physiol Genomics 27: 219-230.

2. Boom R, Sol CJA, Salimans MMM, Jansen CL, Wertheimvandillen PME, et al. (1990) Rapid and Simple Method for Purification of Nucleic-Acids. Journal of Clinical Microbiology 28: 495-503.
